# A review of studies of the proteomes of circulating microparticles: key roles for galectin-3-binding protein-expressing microparticles in vascular diseases and systemic lupus erythematosus

**DOI:** 10.1186/s12014-017-9146-0

**Published:** 2017-04-08

**Authors:** Christoffer T. Nielsen, Ole Østergaard, Niclas S. Rasmussen, Søren Jacobsen, Niels H. H. Heegaard

**Affiliations:** 1 Copenhagen Lupus and Vasculitis Clinic, Centre for Rheumatology and Spine Diseases, Rigshospitalet, Copenhagen University Hospital, Blegdamsvej 9, 2100 Copenhagen Ø, Denmark; 2 Department of Autoimmunology and Biomarkers, Statens Serum Institut, Artillerivej 5, 2300 Copenhagen, Denmark; 3 Department of Clinical Biochemistry and Pharmacology, Odense University Hospital, University of Southern Denmark, Søndre Boulevard 29, 5000 Odense, Denmark

**Keywords:** Proteomics, Mass spectrometry, Microparticles, Systemic lupus erythematosus, Lupus nephritis, Venous thrombosis, Atherosclerosis, Galectin-3-binding protein, Mac-2 binding protein, Alpha-2-macroglobulin, CD5 antigen-like protein

## Abstract

Subcellular microvesicles (MVs) have attracted increasing interest during the past decades. While initially considered as inert cellular debris, several important roles for MVs in physiological homeostasis, cancer, cardiovascular, and autoimmune diseases have been uncovered. Although still poorly understood, MVs are involved in trafficking of information from cell-to-cell, and are implicated in the regulation of immunity, thrombosis, and coagulation. Different subtypes of extracellular MVs exist. This review focuses on the cell membrane-derived shedded MVs (ranging in size from 200 to 1000 nm) typically termed microparticles (MPs). The numbers and particularly the composition of MPs appear to reflect the state of their parental cells and MPs may therefore carry great potential as clinical biomarkers which can be elucidated and developed by proteomics in particular. Determination of the identity of the specific proteins and their quantities, i.e. the proteome, in complex samples such as MPs enables an in-depth characterization of the phenotypical changes of the MPs during disease states. At present, only a limited number of proteomic studies of circulating MPs have been carried out in healthy individuals and disease populations. Interestingly, these studies indicate that a small set of MP-proteins, in particular, overexpression of galectin-3-binding protein (G3BP) distinguish MPs in patients with venous thromboembolism and the systemic autoimmune disease, systemic lupus erythematosus (SLE). G3BP is important in cell–cell adhesion, clearance, and intercellular signaling. MPs overexpressing G3BP may thus be involved in thrombosis and hemostasis, vascular inflammation, and autoimmunity, further favoring G3BP as a marker of “pathogenic” MPs. MPs expressing G3BP may also hold a potential as biomarkers in other conditions such as cancer and chronic viral infections. This review highlights the methodology and results of the proteome studies behind these discoveries and places them in a pathophysiological and biomarker perspective.

## Introduction: microvesicles–microparticles

Microvesicles (MVs) are a heterogenous population of submicron membranous vesicles released from all types of cells both constitutively and during activation and apoptosis [1]. MVs are released from cytosolic organelles, such as granules, from the endoplasmic reticulum, and from multivesicular bodies and are also shed directly from the cell surface membrane [1]. Even though the field is rapidly growing, the limited knowledge about the different types of MVs, their formation, composition, fate, and function still hampers the definitions, terminology, and studies. The MVs that derive from the multivesicular bodies are termed exosomes and range in size from 40 to 100 nm, while the larger MVs (200–1000 nm) shed from the cell surface are typically termed microparticles (MPs) [1]. In this review, we focus on human MP studies based on this definition.

MPs are composed of a complex mixture of proteins, glycoproteins, lipids, and RNA/DNA [2]. They carry many bioactive molecules, and their structure which is reminiscent of liposomes enhances their co-stimulatory properties similar to adjuvants in vaccines [3]. MPs interact with other cells, platelets, other MPs, and the extracellular matrix and facilitate intercellular communication. As examples, MPs can shuttle mRNA and miRNA to acceptor cells and regulate their protein translation, MPs serve as a major mobilizable reservoir of tissue factor (TF) and can initiate coagulation, and MPs can alter immune responses in autoimmune diseases, e.g. by triggering dendritic cells to release interferon-α (IFN-α), a major effector cytokine in systemic lupus erythematosus (SLE) [1, 4, 5]. Most functional studies have been conducted in vitro by investigating subsets of MPs or specific surface molecules from MPs induced in cultured cells [6]. Functional MP studies ex vivo have been few [5]. Instead, investigations in patients have focused on quantitating cellular subsets of circulating MPs and correlating them to a specific disease or disease manifestation and thereby explore their pathogenic roles and/or biomarker potential [7]. Generally, MPs increase in numbers during cellular activation or apoptosis, but determining their numbers and origin have not proved specific or sensitive enough for biomarker use. More extensive and detailed characterization of the specific MP subpopulations is highly needed. Until now most studies have characterized MP subpopulations using cell-specific surface markers due to the paucity of other identified markers specific for subsets of MPs involved e.g. in pathological processes [8–10].

Unbiased in-depth characterization of DNA/RNA content, lipids, metabolites, and proteins may help unravel the MP-composition and the discovery of new markers of MPs that are involved in disease pathogenesis. Here, we review the methodology and results of proteomic discovery studies of human plasma MPs. We argue that a restricted set of candidate MP proteins, galectin-3-binding protein (G3BP) in particular, could prove highly useful as biomarkers and tools to understanding the role of MPs in the systemic autoimmune disease, systemic lupus erythematosus (SLE), and venous thromboembolism (VTE), and potentially also in cancer and chronic viral infections [8, 11–15]. G3BP is a cysteine-rich scavenger receptor [16, 17]. G3BP exerts its regulatory roles by binding to lectins, extracellular matrix proteins, and integrins [18–20]. G3BP is upregulated during cell activation, viral infections, cell death, and in cancer cells, and G3BP-expression could reflect an aberrant state of the parental cells, e.g. apoptosis [21–24]. In HIV and hepatitis C, serum G3BP has been associated with a poorer outcome [22, 25–28]. G3BP plays important roles in cell–cell adhesion and intercellular signaling in the immune system and in cancer and metastasis [29–32]. Breast cancer cells in vitro release G3BP which promotes tumor cell aggregation and inhibition of peri-tumor fibrosis ultimately favoring metastasis [31, 33, 34]. In line with these observations, serum G3BP is associated with a poorer prognosis in non-Hodgkin lymphoma, lung and breast cancer [33, 35, 36]. In this review we focus on G3BP-expressing MPs in SLE and VTE. However, G3BP may hold far greater potential as a novel MP-biomarker in other conditions.

## Microparticle proteomics

### Proteome strategies and methods

The design, pre-analytical and analytical methodology, and the results of the circulating human MP proteome studies to date are presented in detail in Table 1. In all cases the MPs were isolated by centrifugation from plasma, in some cases combined with prior size exclusion chromatography. It must be noted, however, that the exact conditions (to the extent they are specified) differ, i.e. different anticoagulants and—in particular—different centrifugation conditions (time, temperature, volumes, and g-forces) were used. This makes it difficult to compare studies, even those that aim to characterize and establish MP proteomes of healthy individuals [37–40]. So far, only one study has attempted a systematic optimization of MP isolation steps monitored by objective parameters such as number and reproducibility of protein identifications [40]. The incomparability of data between studies of circulating MPs in normal controls is compounded by the vastly different analytical conditions and data extraction methods (Table 1). Many studies rely on liquid chromatography–tandem mass spectrometry (LC–MS/MS) with no labeling and other studies use isobaric tags for relative and absolute quantitation (iTRAQ) or isotope-coded affinity tags (ICAT). Further, some studies use 1-D or 2-D gel electrophoresis as primary separation and then MALDI-qTOF MS or other MALDI-TOF configurations. The resulting number of identified proteins in normal circulating MPs consequently varies considerably from 151 [12] to more than 530 [11] and contrasts to the more than 1100 common proteins identified in studies on platelet MPs [41]. Also, only rudimentary information on reproducibility within and between runs is available. Further, no reference ranges of MP numbers, types and protein abundance have been established in any population or patient group so far. Every study is a compromise between how extensively (how deeply) MP proteomes are characterized and the analysis time required for this weighed against the number of samples. This may favor pooling of samples in some cases but this should only be done after careful consideration of pros and cons [42]. Due to the complexity and heterogeneity of the MP compartment in blood samples some studies concentrate on the MPs produced by platelets, erythrocytes, or granulocytes. This is accomplished either by culturing the cells and studying the MPs they release after various stimuli or by isolating (e.g. by immunoaffinity methods) the specific MPs from plasma by virtue of their surface markers. By far the largest group of this type of studies deal with platelet-MPs derived from platelets isolated by centrifugation and then subjected to stimuli such as ADP, thrombin, and/or collagen [11–16]. Platelet MPs are normally among the most numerous in the circulation and are important in cardiovascular events both in venous and arterial thrombosis. Characterization of the ensemble of circulating MPs in plasma is addressed wholly or partly in eight of the studies included in Table 1 [17–24].

**Table 1:** Proteome studies of circulating microparticles, and of in vitro generated platelet-and granulocyte-derived microparticles. Study design, methodology and main results

Study	References	Objectives	Study population^a^	Pre-analytical protocol	Details	Proteome analysis workflow	Details	Main findings
Platelet-derived MPs	Garcia et al. [49]	Characterize and compare the proteome of in vitro generated platelet-derived MPs (PMPs) to platelets	Individuals: 1 healthy donor	Sample collection	Whole-blood Anticoagulant: acid-citrate-dextrose (ACD)	Workflow	Gel-LC–MSMS shotgun workflow: 1-D gel electrophoresis (Coomassie Blue staining) with lanes cut into 26 bands and digested before LC–MS/MS	578 proteins in total were identified in PMPs. 380 of these proteins had not previously been identified in platelets (at the time of publication)
			Sex: not stated	PRP generation (cell removal)	Cent. 110 g/15 min/T?	Tryptic digestion	In-gel digestion	
			Age: not stated	Plt isolation	Cent. 710 g/15 min/T? Followed by 3× wash (Cent. 2000 g/10 min/T?)	Method	Nano-LC–MS/MS	
			Ethnicity: not stated	Plt resuspension before activation	Tyrode’s buffer (with 1.5 mM CaCl_2_, 0.4 mM MgCl_2_); debris and cell removal from plt prep using an additional cent. 110 g/10 min/T?	Instrument	Finnigan LTQ Ion Trap (Thermo Fisher Scientific)	
				Plt activation for PMP in vitro generation	ADP	Software for protein ID	SEQUEST	
				Removal of activated Plts and cells	Cent. 710 g/15 min/T? Followed by 3× wash (Cent. 2000 g/10 min/T?)	Protein database	NCBInr (human) Taxonomy: human # Entries: not stated	
				PMP isolation	Cent. 150,000 g/90 min/10 °C (Pellet = MPs)			
Platelet-derived MPs	Piersma et al. [51]	Characterize the releasate (including MPs) from in vitro activated platelets	Individuals: 3 healthy donors	Sample collection	Whole-blood Anticoagulant: acid-citrate-dextrose (ACD) Prostaglandin E1 was added to inhibit platelet activation	Workflow	Gel-LC–MSMS shotgun workflow: 1-D gel electrophoresis (Coomassie staining) with lanes cut into 15 bands (Exp #1) or into 10 bands (Exp #2) and digested before LC–MS/MS	716 proteins in total were identified in the platelet releasate 225 proteins were identified in the releasate from all 3 individuals and defined as the “core” releasate proteome 55% of the “core” proteome overlapped with previous publications of PMP and α-granule proteomes 45% of the “core” proteome was unique to the platelet realesate The unstimulated control showed few bands on SDS-PAGE gel and was not analysed
			Sex: not stated	PRP generation (cell removal)	Cent. 150 g/15 min/RT	Tryptic digestion	In-gel digestion	
			Age: not stated	Plt isolation	Cent. 720 g/10 min/RT Followed by 2× wash in Tyrode’s buffer	Method	Nano-LC–MS/MS	
			Ethnicity: not stated	Plt resuspension before activation	Tyrode’s buffer	Instrument	LTQ-FT hybrid mass spectrometer (Thermo Fisher Scientific)	
				Plt activation for PMP in vitro generation	Thrombin receptor activating peptide, non-stimulated platelets as controls	Software for protein ID	SEQUEST	
				Removal of activated Plts and cells	2× Cent. 1000 g/10 min	Protein database	IPI.human v3.31 Taxonomy: human # Entries: 67,511	
				Plt releasate isolation	Supernatant from plt removal was concentrated on Amicon Ultra-4 cell (10 kDa cut-off)			
Platelet-derived MPs	Dean et al. [52]	Characterize the proteome of in vitro generated platelet-derived MPs of different sizes	Individuals: 7–10 healthy donors	Sample collection	Whole-blood Anticoagulant: citrate Samples were pooled before plt isolation	Workflow	Shot-gun proteomics workflow	Total number of identified proteins based on size-exclusion fractions: 54 (#1), 49 (#2), 293 (#3), and 150 (#4) Proteins were highly differentially expressed between the four fractions Mitochondrial proteins were enriched in fraction 1 Cytoskeletal proteins were enriched in fractions 3 and 4 and more similar to plasma membranes
			Sex: not stated	PRP generation (cell removal)	2× Cent. 150 g/10 min/RT	Tryptic digestion	In-solution digestion	
			Age: not stated	Plt isolation	2× Cent. 1500 g/10 min/RT	Method	2D-LC–MS/MS	
			Ethnicity: not stated	Plt resuspension before activation	Tyrode’s buffer (calcium free)	Instrument	LTQ Plus Ion Trap (Thermo Fisher Scientific)	
				Plt activation for PMP in vitro generation	CaCl_2_, thrombin, collagen	Software for protein ID	SEQUEST	
				Removal of activated Plts and cells	Cent. 5000 g/t?/T?	Protein database	Human Ref-Seq Taxonomy: Human # Entries: not stated	
				PMP isolation	Cent. 130,000 g/t?/T? (Pellet = MPs) Pellet were then subject to gel-filtration to obtain 4 different PMP size fractions			
Platelet-derived MPs	Shai et al. [46]	Characterize in vitro generated platelet-derived MP proteomes according to different platelet activation stimuli	Individuals: 4 healthy donors	Sample collection	Fresh platelets were collected using an apheresis system (MCS platelet collection system) and washed (solution containing citric acid, PGE1 and apyrase)	Workflow	2-D gel electrophoresis (Sypro Ruby staining), differential image analysis to identify differentially expressed proteins and ID of spots of interest using MS	26 proteins were identified as differentially expressed between thrombin induced and shear-stress induced MPs
			Sex: not stated	PRP generation (cell removal)	See sample collection	Tryptic digestion	In-gel digestion	
			Age: 20–35 yrs	Plt isolation	See sample collection	Method	MALDI-TOF/TOF or LC–MS/MS	
			Ethnicity: not stated	Plt resuspension before activation	Buffer not stated	Instrument	4800 MALDI-TOF/TOF (Applied Biosystems) Amazon ETD Ion Trap (Bruker)	
				Plt activation for PMP in vitro generation	Thrombin or shear stress	Software for protein ID	Mascot v2.1 and Mascot v2.3	
				Removal of activated Plts and cells	2× Cent. 1500 g/t?/T?	Protein database	SwissProt release 56.0 Taxonomy: Human Entries: not stated SwissProt release 57.15 Taxonomy: Human # Entries: not stated	
				PMP isolation	Cent. 100,000 g/60 min/T? (Pellet = MPs)			
Platelet-derived MPs	Capriotti et al. [56]	Comparative analysis of the proteome of in vitro generated PMPs using a shot-gun proteomic protocol to analyze in-solution digested proteins obtained with or without an additional hydrogel nanoparticle (HN) enrichment protocol for low molecular weight proteins	Individuals: 12 healthy donors	Sample collection	Whole blood. Anti-coagulant: K2EDTA and protease inhibitors cocktail. Samples were pooled before Plt isolation	Workflow	Shot-gun proteomics workflow	In total 603 proteins were identified combining identifications obtained with or without the HN enrichment step 318 proteins were exclusively identified using the standard procedure and 57 proteins were only identified by including the HN enrichment step, while the remaining 228 proteins were identified using both methods A higher proportion of low molecular weight proteins were found in the HN protocol Compared to the PMP proteome in Garcia et al. (2005), 360 proteins overlapped, while 243 did not overlap. Of the 243 proteins, 130 had previously been confined to the platelet proteome
			Sex: All males	PRP generation (cell removal)	Cent. 110 g/15 min/T?	Tryptic digestion	Proteins precipitated using chloroform/methanol method before in-solution digestion	
			Age: 20–40 yrs	Plt isolation	Cent. 710 g/15 min/T? Followed by 3× wash (Cent. 2000 g/10 min)	Method	Nano-LC–MS/MS	
			Ethnicity: not stated	Plt resuspension before activation	Tyrode’s buffer (with 1.5 mM CaCl_2_, 0.4 mM MgCl_2_); debris and cell removal from plt prep using an additional cent. 110 g 10 min	Instrument	LTQ-Orbitrap XL (Thermo Fisher Scientific)	
				Plt activation for PMP in vitro generation	ADP	Software for protein ID	Proteome Discoverer v1.2 and Mascot v 2.3.2 Protein IDs validated in Scaffold v3.1.2	
				Removal of activated Plts and cells	Cent. 710 g/15 min/T?	Protein database	SwissProt release 57.15 Taxonomy: Human # Entries: 20,266	
				PMP isolation	Cent. 150,000 g/90 min/4 °C (Pellet = MPs)			
Platelet-derived MPs	Milioli et al. [41]	Quantative proteome analysis of in vitro generated platelet MPs formed by platelet activation using agonists of increasing potency	Individuals: 3 healthy donors	Sample collection	Fresh platelets were collected using an apheresis system	Workflow	Shot-gun proteomics workflow, Differentially expressed proteins identified using the iTRAQ method	3383 proteins were identified 1109 proteins were identified in all three biological replicates Significant differential expression of proteins dependent on the stimuli was observed
			Sex: not stated	PRP generation (cell removal)	See above	Tryptic digestion	In-solution digestion	
			Age: not stated	Plt isolation	Cent. 700 g/20 min/20 °C Followed by 2× wash in Tyrode’s buffer (with 1 mM EDTA)	Method	iTRAQ labelleing of peptides was followed by HILIC fractionation before analysis by nano-LC–MS/MS	
			Ethnicity: not stated	Plt resuspension before activation	Tyrode’s buffer (pH 6)	Instrument	Q-Exactive Plus (Thermo Fisher Scientific)	
				Plt activation for PMP in vitro generation	(a) 10 uM ADP (b) 1 U/mL thrombin (c) 20 ug/mL collagen (d) 1 U/mL thrombin and 20 mg/mL collagen	Software for protein ID	Proteome Discoverer v1.4.0.288 in combination with Mascot v2.3 and SEQUEST HT	
				Removal of activated Plts and cells	Cent. 710 g/20 min/20 °C Cent. 1000 g/20 min/20 °C	Protein database	SwissProt release v3.53 Taxonomy: Human # Entries: 20,243	
				PMP isolation	Cent. 130,000 g/60 min/4 °C (Pellet = MPs)			
Plasma MPs	Little et al. [39]	Characterize the plasma MP proteome in a general population	Individuals: 42 patients diagnosed with: (a) Cardiovascular disease (b) Hypertension (c) Diabetes mellitus (d) Cancer	Sample collection	Whole-blood Anti-coagulant: acid-citrate-dextrose	Workflow	Shot-gun proteomics workflow	458 proteins were identified 130 proteins were identified in 50% of the samples and defined as the “core” proteome Both “core” and non-”core” protein expression were correlated to age and gender. Correlates to comorbidities were not stated
			Age: 61–77 yrs (avg 69.5 yrs)	PRP generation (cell removal)	Cent. Unknown g/15 min/RT	Tryptic digestion	In-solution digestion	
			Sex: 12 female, 30 males	PPP/PFP generation (Plt removal)	2× Cent. 2000 rpm (Unknown g)/15 min/RT	Method	Nano-LC–MS/MS	
			Ethnicity: 40 Caucasian, 2 African-American	Storage of PPP/PFP before MP isolation	Immediate processing of PPP to isolate MPs	Instrument	LTQ-FT hybrid mass spectrometer (Thermo Fisher Scientific)	
			Other: 7 Smokers	MP isolation	Gel filtration (not further specified) followed by cent. 100,000 g/120 min/T?	Software for protein ID	SEQUEST	
						Protein database	IPI.human (version not stated) Taxonomy: human # Entries: not stated	
Plasma MPs	Ramacciotti et al. [12]	Characterize the plasma MP proteome in patients with deep venous thrombosis (DVT) compared to controls	Individuals: (a) 9 patients with DVT (b) 9 patients with leg pain without DVT (c) 6 healthy controls	Sample collection	Whole-blood Anti-coagulant: acid-citrate-dextrose	Workflow	Shot-gun proteomics workflow, Differentially expressed proteins identified using the iTRAQ method	151 proteins were identified 35 proteins displayed enrichment or depletion in plasma MPs in DVT patients in two out three experiments Alpha-2-macroglobulin and galectin-3-binding protein (G3BP) were the only enriched proteins in all DVT patients 11 proteins were depleted in all three experiments in plasma MPs in DVT patients
			Sex: not stated	PRP generation (cell removal)	NA (PPP was generated directly from whole blood)	Tryptic digestion	In-solution digestion	
			Age: not stated	PPP/PFP generation (Plt removal)	Cent. 1500 g/25 min/RT followed by 2× Cent. 15,000 g/2 min/RT resulting in PFP	Method	iTRAQ labelleing of peptides was followed by SCX fractionation before analysis by nano-LC–MS/MS	
			Ethnicity: not stated	Storage of PPP/PFP before MP isolation	PFP was stored at −70 °C (12–24 mo) before MP isolation	Instrument	4800 MALDI-TOF/TOF (Applied Biosystems)	
				MP isolation	Cent. 200,000 g/120 min/4 °C Followed by 1× wash in 0.25 M KBr and Cent. 200,000 g/120 min/4 °C	Software for protein ID	GPS Explorer v3.6 in combination with Mascot v2.1	
						Protein database	NCBInr Taxonomy: mammals # Entries: not stated	
Plasma MPs	Ostergaard et al. [40]	Characterize the plasma MP proteome. Evaluate the MP washing procedure, reproducibility, and compare analysis of pooled to individual samples	Individuals: 12 healthy donors	Sample collection	Whole blood Anti-coagulant: sodium citrate	Workflow	Shot-gun proteomics workflow, differentially expressed proteins identified by label-free quantitation	536 proteins were identified High analytical reproducibilty Significant overlap between pools and single samples 334 proteins were identified in 50% of the samples and were defined as the healthy “core” proteome Four washing steps were needed to purifiy plasma MPs
			Sex: 8 female, 4 male	PRP generation (cell removal)	Cent. 1800 g/10 min/21 °C	Tryptic digestion	In-solution digestion	
			Age: 24–62 yrs (avg 41.1 yrs)	PPP/PFP generation (Plt removal)	Cent. 3000 g/10 min/21 °C	Method	Nano-LC–MS/MS	
			Ethnicity: All Caucasian	Storage of PPP/PFP before MP isolation	PPP was stored at −80 °C before MP isolation	Instrument	LTQ-Orbitrap XL (Thermo Fisher Scientific)	
				MP isolation	Cent. 18,890 g/30 min/21 °C followed by 4× wash in PBS-citrate (154 mM NaCl, 1.4 mM phosphate, 10.5 mM trisodium citrate, pH 7.4) and 4X Cent. 18,890 g/30 min/21 °C (Pellet = MPs)	Software for protein ID	MaxQuant v1.1.1.25 with the built-in Andromeda search engine	
						Protein database	IPI.human.v3.68.fasta Taxonomy: human # Entries: 87,061	
Plasma MPs	Bastos-Amador et al. [48]	Characterize the plasma MP proteome from healthy controls	Individuals: 38 healthy donors	Sample collection	Not stated (Plasma was obtained from a blood bank)	Workflow	Shot-gun proteomics workflow	161 microparticle-associated proteins were identified 52 of the identified proteins belong to the immunoglobulin family Large variability was observed in the protein complement identified from MPs isolated from different donors
			Sex: 11 males, 27 females	PRP generation (cell removal)	Not stated	Tryptic digestion	In-solution digestion	
			Age: 20–58 yrs (avg 40.9 yrs)	PPP/PFP generation (Plt removal)	Cent. 2000 g/30 min/T? followed by Cent. 12,000 g/45 min/T?	Method	Nano-LC–MS^E^	
			Ethnicity: not stated	Storage of PPP/PFP before MP isolation	Immediate processing of PFP to isolate MPs	Instrument	Q-Tof Premier (Waters Corporation)	
				MP isolation	Cent. 110,000 g/120 min/T? followed by Filtering 0.22 um followed by Cent. 100,000 g/60 min/T? (Pellet = MPs)	Software for protein ID	ProteinLynx GlobalServer v 2.6	
						Protein database	SwissProt (version not stated) Taxonomy: not stated # Entries: not stated	
Plasma MPs	Chaichompoo et al. [54]	Characterize plasma MPs in patients with β-thalassemia/hemoglobin E and healthy controls	Individuals: (a) 15 β-thalassemia/hemoglobin E patients (β-thal/HbE) (b) 15 healthy donors	Sample collection	Whole blood Anti-coagulant: K3EDTA, heparin, citrate	Workflow	2-D gel electrophoresis with silver staining Differentially expressed proteins selected by image analysis (Image Master Platinum) followed by identification by mass spectrometry analysis	1000–1200 proteins spots were identified in each 2-D gel 29 protein spots differed significantly 9 proteins were decreased in β-thal/HbE patients 12 proteins were found increased in β-thal/HbE patients 8 proteins were only detected in MPs β-thal/HbE patients
			Sex: not stated	PRP generation (cell removal)	Cent. 1500 g/15 min/20 °C	Tryptic digestion	In-gel digestion	
			Age: 30.9 ± 8.9 yrs (patients) Age: 25.3 ± 1.8 yrs (healthy donors)	PPP/PFP generation (Plt removal)	2× Cent. 14,000 g/2 min/20 °C resulting in PFP	Method	MALDI Q-TOF	
			Ethnicity: All asians	Storage of PPP/PFP before MP isolation	Immediate processing of PFP to isolate MPs	Instrument	MALDI Q-TOF Ultima (Micromass)	
				MP isolation	Cent. 14,000 g/45 min/20 °C followed by 2× wash in PBS (with 0.32% citrate) and 2× Cent. 14,000 g/10 min/20 °C (Pellet = MPs)	Software for protein ID	Mascot	
						Protein database	NCBInr Taxonomy: not stated (All?) # Entries: 17,172,511	
Plasma MPs	Ostergaard et al. [11]	Characterize the plasma MP proteome in patients with systemic lupus erythematosus (SLE), rheumatoid arthritis (RA), systemic sclerosis (SSc) and healthy controls (NOR)	Individuals: (a) 12 SLE patients (b) 6 RA pateints (c) 6 SSc patients (d) 12 NOR	Sample collection	Whole blood Anti-coagulant: sodium citrate	Workflow	Shot-gun proteomics workflow, differentially expressed proteins identified by label-free quantitation	534 proteins were identified in total 314 of the proteins were idenitified in 2/3 of the samples 191 proteins were twofold upregulated in SLE, in particular: G3BP, ficolin-2, 14-3-3, β-2-glycoprotein-I, and CD5 antigen-like protein, Immunglobulins and complement proteins 57 proteins were twofold decreased in SLE
			Sex (a) SLE: 12 females (b) RA: 2 males, 4 females (c) SSc: 6 females (d) NOR: 4 males, 8 females	PRP generation (cell removal)	Cent. 1800 g/10 min/21 °C	Tryptic digestion	In-solution digestion	
			Age (a) SLE: 23–52 yrs (median 34 yrs) (b) RA: 35–56 yrs (median 39 yrs) (c) SSc: 33–68 yrs (median 45 yrs) (d) NOR: 24–6 yrs (median 35 yrs)	PPP/PFP generation (Plt removal)	Cent. 3000 g/10 min/21 °C	Method	Nano-LC–MS/MS	
			Ethnicity: All Caucasian	Storage of PPP/PFP before MP isolation	PPP was stored at −80 °C before MP isolation	Instrument	LTQ-Orbitrap XL (Thermo Fisher Scientific)	
				MP isolation	Cent. 18,890 g/30 min/21 °C followed by 4× wash in PBS-citrate (154 mM NaCl, 1.4 mM phosphate, 10.5 mM trisodium citrate, pH 7.4) and 4X Cent. 18,890 g/30 min/21 °C (Pellet = MPs)	Software for protein ID	MaxQuant v1.1.1.36 with the built-in Andromeda search engine	
						Protein database	IPI.human.v3.68.fasta Taxonomy: Human # Entries: 87,061	
Plasma MPs	Datta et al. [55]	Compare the plasma MP proteome in subgroups of patients with lacunar infarction (LACI) and controls	Individuals: (a) 45 LACI patients: Group I: no adverse events, n = 19 Group II: recurrent vascular events, n = 11 Group III: cognitive decline, no recurrent vascular events, n = 15 (b) 17 healthy controls	Sample collection	Whole blood Anti-coagulant: EDTA	Workflow	Gel-LC–MSMS shotgun workflow: 1-D gel electrophoresis (Stain not stated) with lanes cut into bands (Number not stated) and digested before LC–MS/MS. Differentially expressed proteins identified using the iTRAQ method	183 proteins were identified 43 specific proteins were overexpressed compared to the healthy controls: Group I: 17, Group II: 33, Group II: 28 Of the 43 proteins myelin basic protein were most upregulated Cluster analysis revealed two groups of proteins associated with clinical outcome G3BP was decreased and together with A2M were not associated with any of the disease groups
			Sex (a) LACI patients Group I: 17 males, 2 females Group II: 8 males, 3 females Group III: 5 males, 10 females (b) Controls: 4 males, 22 females	PRP generation (cell removal)	Conditions for PRP generation not stated PRP was frozen before further work	Tryptic digestion	In-gel digestion	
			Age (Mean (SD)) (a) LACI patients Group I: 61 yrs (9 yrs) Group II:65 yrs (10 yrs) Group III: 66 yrs (9 yrs) (b) Controls: 56 yrs (9 yrs)	PPP/PFP generation (Plt removal)	Samples were pooled before platelet removal 2× Cent. 4000 g/30 min/T? followed by Cent 12,000 g/30 min/T?	Method	iTRAQ labelleing of peptides was followed by ERLIC fractionation before analysis by nano-LC–MS/MS	
			Ethnicity (a) LACI patients Group I: 17 of 19 Chinese Group II: 8 of 11 Chinese Group III: 15 of 15 Chinese (b) Controls: 17 of 17 Chinese	Storage of PPP/PFP before MP isolation	Immediate processing of PFP to isolate MPs	Instrument	QSTAR Elite Hybrid MS (Applied Biosystems)	
				MP isolation	Cent. 30,000 g/120 min/T? followed by Cent. 200,000 g/135 min/T? (Pellet = MPs) followed by 2× wash with PBS; Cent. not stated	Software for protein ID	ProteinPilot v 3.0	
						Protein database	UniProt - version not stated Taxonomy: Human # Entries 191,242	
Plasma and platelet-derived MPs	Jin et al. [37]	Compare the plasma MP proteome to plasma and platelet proteomes	Individuals: 16 healthy donors	Sample collection	Whole blood Anti-coagulant: sodium citrate	Workflow	2-D gel electrophoresis (Sypro Ruby staining), differential image analysis (ImageMaster 2D) to identify differentially expressed proteins and ID of spots of interest using MS	1021–1055 protein spots were identified in the plasma MPs 331–370 protein spots were identified in plasma (PPP) 169 overexpressed protein spots in plasma MPs were excised for protein identification 83 proteins including their isoforms were identified in plasma MPs 30 of these proteins had not been previously identified in the plasma proteome
			Sex: 8 males, 8 females	PRP generation (cell removal)	Not stated (Plasma samples were obtained frozen)	Tryptic digestion	In-gel digestion	
			Age: 18–65 yrs	PPP/PFP generation (Plt removal)	Cent. 3200 g/30 min/T?	Method	MALDI-TOF/TOF	
			Ethnicity: not stated	Storage of PPP/PFP before MP isolation	Immediate processing of PFP to isolate MPs	Instrument	4700 Proteomics Analyzer (Applied Biosystems)	
				MP isolation	Cent. 250,000 g/60 min/T? (Pellet = MPs) followed by 2× wash with PBS (Cent. not stated)	Software for protein ID	Mascot (version not stated)	
						Protein database	Not stated (SwissProt -derived from ID table) Taxonomy: not stated # Entries: not stated	
Plasma and platelet-derived MPs	Smalley et al. [38]	Compare the plasma MP proteome to in vitro generated platelet-derived MPs	Individuals: 3 healthy donors	Sample collection	Whole-blood Anticoagulant: acid-citrate-dextrose (ACD)	Workflow	Gel-LC–MSMS shotgun workflow 1-D gel electrophoresis (No staining) with lanes cut into 1 bands and digested before LC–MS/MS Differentially expressed proteins identified by spectral counting or using the ICAT labelling method	19 proteins were detected in plasma MPs and not in PMPs 2 proteins (von Willebrand factor and albumin) were detected in both populations but enriched in plasma MPs 11 proteins were enriched in platelet-derived MPs
			Sex: not stated	PRP generation (cell removal)	Cent. 110 g/15 min/T?	Tryptic digestion	In-gel digestion	
			Age: not stated	Plt isolation	Cent. 710 g/15 min/T? (Supernatant = PPP) Followed by 3× wash (Cent. 2000 g/10 min/T?)	Method	Nano-LC–MS/MS	
			Ethnicity: not stated	Plt resuspension before activation	Tyrode’s buffer (with 1.5 mM CaCl_2_, 0.4 mM MgCl_2_); debris and cell removal from plt prep using an additional cent. 110 g/10 min/T?	Instrument	Finnigan LTQ Ion Trap (Thermo Fisher Scientific)	
				Plt activation for PMP in vitro generation	ADP	Software for protein ID	SEQUEST	
				Removal of activated Plts and cells	Cent. 710 g/15 min/T? Followed by 3× wash (Cent. 2000 g/10 min/T?)	Protein database	NCBInr (Human) Taxonomy: Human # Entries: not stated	
				PMP isolation	Cent. 150,000 g/90 min/10 °C (Pellet = MPs)			
				Sample collection	Whole-blood. Anticoagulant: acid-citrate-dextrose (ACD)			
				PRP generation (cell removal)	Cent. 110 g/15 min/T?			
				PPP/PFP generation (Plt removal)	Cent. 710 g/15 min/T? (Sup. = PPP) followed by 2× Cent. 710 g/15 min/25 °C			
				Storage of PPP/PFP before MP isolation	Immediate processing of PPP to isolate MPs			
				MP isolation	Gelfiltration of PPP followed by cent. 150,000 g/90 min/10 °C (Pellet = MPs)			
Erythrocyte-derived MPs	Rubin et al. [50]	Characterize the proteome of MPs derived from erythrocyte concentrates (ECs) to explore the effect of blood storage	Individuals: number of donors not stated	Sample collection	Whole-blood (collected in 500 ml in blood bags) Anticoagulant: citrate–phosphate-dextrose Leukocytes and platelets were removed by filtration Plasma was removed from erythrocytes by centrifugation (conditions not stated) Erythrocytes were resuspended in sodium-adenine-glucose-mannitol solution and stored as erythrocyte concentrates (ECs) at 4 °C until further analysis	Workflow	1-D gel electrophoresis (Coomassie Blue staining); bands of interest were out and in-gel digested MS analysis	24 proteins were identified from selected bands from an SDS-PAGE gel lane loaded with erythrocyte membranes and 16 proteins were identified from a lane loaded with erythrocyte derived MPs Carbonic anhydrases, peroxiredoxins, and 14-3-3 proteins were abundant but not differentially expressed between membranes and MPs Stomatin, Band 3 and Rhesus protein were enriched in erythrocyte MPs
			Sex: not stated	PRP generation (cell removal)	Not applicable	Tryptic digestion	In-gel digestion	
			Age: not stated	PPP/PFP generation (Plt removal)	Not applicable	Method	MALDI-TOF/TOF or LC–MS/MS	
			Ethnicity: not stated	Storage of PPP/PFP before MP isolation	Not applicable	Instrument	4700 MALDI-TOF/TOF (Applied Biosystems)	
				MP isolation	2× Cent. 1850 g/20 min/4 °C (of ECs) followed by Cent. 3200 g/20 min/4 °C followed by 3× Cent. 120,000 g/90 min/4 °C (Pellet = MPs)	Software for protein ID	Mascot v2.0	
						Protein database	UniProt - version not stated Taxonomy: not stated # Entries: not stated	
Erythrocyte-derived MPs	Bosman et al. [53]	Compare the proteome of erythrocyte-derived plasma MPs to erythrocyte membranes	Individuals: number of donors not stated	Sample collection	Whole-blood Anticoagulant: citrate	Workflow	Gel-LC–MSMS shotgun workflow: 1-D gel electrophoresis (Coomassie Blue staining) with lanes cut into 6 bands and digested before LC–MS/MS. Differentially expressed proteins were identified using the emPAI method	271 proteins were identified in erythrocyte-derived MPs and erythrocyte membrane fractions 71 different proteins were identified in erythrocyte-derived MPs Comparison of the differential distribution of proteins based on their subcellular localization/function
			Sex: not stated	PRP generation (cell removal)	Cent. 1550 g/7 min/20 °C	Tryptic digestion	In-gel digestion	
			Age: not stated	PPP/PFP generation (Plt removal)	Cent. 1550 g/7 min/20 °C	Method	Nano-LC–MS/MS	
			Ethnicity: not stated	Storage of PPP/PFP before MP isolation	Immediate processing of PPP to isolate MPs	Instrument	LTQ-FT hybrid mass spectrometer (Thermo Fisher Scientific)	
				MP isolation	2× Cent. 40,000 g/20 min/4 °C followed by Flow assisted cell sorting to isolate erythrocyte and platelet derived MPs.	Software for protein ID	Mascot v 2.1	
						Protein database	NCBInr (Human) Taxonomy: Human # Entries: not stated	
Granulocyte-derived MPs	Dalli et al. [10]	Determine MP proteomic changes due to different granulocyte stimuli, identify candidate granulocyte-derived MP biomarkers, and test a panel of identified MP-biomarkers in sepsis patients and controls	Individuals: (a) 16 healthy donors (b) 10 donors with blister exudate (c) 10 donors with sepsis	Sample collection	Whole-blood Anticoagulant: not stated	Workflow	1-D gel electrophoresis (Silver staining); bands of interest were out and in-gel digested MS analysis Differentially expressed proteins were identified by using normalized spectral counts	342 proteins could be identified from MPs derived in vitro from neutrophils stimulated in immobilized phase (post adhesion to HUVECs) 302 proteins were identified from MPs derived in vitro from neutrophils stimulated in fluid phase 30% of the proteins were uniquely expressed in one of the two MP classes A2M and ceruloplasmin were particular enriched in the immobilized phase induced MPs, whereas heat shock 70 kDa protein 1 was enriched in fluid phase induced MPs, and Annexin A1 was expressed equally between the two classes of MPs Granulocyte-derived MPs from plasma showed—using flow cytometry—significantly different expression of these proteins between septic patients, healthy controls and exudate MPs
			Sex: (a) 9 males and 7 females (b) 6 males and 4 females (c) 2 males and 8 females	PRP generation (cell removal)	Cent. 1600 g/10 min/4 °C	Tryptic digestion	In-gel digestion	
			Age: (a) 30.0 ± 0.5 yrs (b) 31.3 ± 0.4 yrs (c) 58.8 ± 1.6 yrs	PPP/PFP generation (Plt removal)	Not performed	Method	Nano-LC–MS/MS	
			Ethnicity: not stated	Storage of PPP/PFP before MP isolation	PRP was stored at −80 °C before MP isolation	Instrument	LTQ-Orbitrap XL (Thermo Fisher Scientific)	
				MP isolation	2× Cent. 3000 g/10 min/4 °C followed by Cent. 100,000 g/60 min/4 °C (Pellet = MPs) Isolated MPs were frozen at −80 °C before analysis	Software for protein ID	SEQUEST v28 in combination with X!Tandem v2007.01.01.2. In addition Scaffold v2.0.5 was used for peptide and protein validation and for spectral count calculation	
				In vitro generated granulocyte-derived MPs	Granulocytes were isolated from the buffy coat using Ficoll-Hypaque. Granulocytes were stimulated by fMLF activation (fluid phase) or incubation with a HUVEC monolayer (solid phase). MP isolation: centrifugation by 3000 g/10 min/4 °C twice, then 100,000 g/60 min/4 °C and frozen at −80 °C	Protein database	UniProtKB/SwissProt v14.6 Taxonomy: Human # Entries: 20,333	
				Exudate MPs	Blisters were induced by cantharidin and exudates were harvested after 24 h, MP isolation not stated			

Tyrode’s buffer: buffer composed of 8.0 g/L NaCl, 0.2 g/L KCl, 0.20 g/L CaCl2, 0.10 MgCl2, 0.05 g/L NaH2PO4; 1.0 g/L NaHCO3, 1.0 g/L Glucose with minor variations

*ACD* acid-citrate-dextrose, *ADP* adenosine diphosphate, *Avg* average, *Cent* centrifugation, *DVT* deep venous thrombosis, *EC* erythrocyte concentrates, *EDTA* ethylenediaminetetraacetic acid, *emPAI* exponentially modified Protein Abundance Index, *ERLIC* electrostatic repulsion and hydrophobic interaction chromatography, *Exp* experiment, *fMLF* formyl-methionyl-leucyl phenylalanine, *HILIC* hydrophobic interaction liquid chromatography, *HN* hydrogel nanoparticles, *HUVEV* human umbilical cord endothelial cells, *LACI* lacunar infarction, *LC* liquid chromatography, *Mo* months, *MP* microparticles, *MS* mass spectrometry, *NA* not applicable, *nano*-*LC*-*MS/MS* nano-flow liquid chromatography coupled tandem mass spectroscopy, *PFP* platelet free plasma, *Plt* platelets, *PMP* Platelet-derived microparticle, *PPP* platelet poor plasma, *PRP* platelet rich plasma, *RA* rheumatoid arthritis, *RT* room temperature, *SCX* strong cation exchange, *SLE* systemic lupus erythematosus, *SSc* systemic sclerosis, *T?* temperature not stated, *t?* time not stated, *TRAP* thrombin receptor activating peptide, *Yrs* years

Age is the age (mean, median, SD, and/or range) stated in the article

### The microparticle protein composition reflects the state of the parental cell

Since the composition of MPs appears to reflect the state of the parental cell, the actual biomarker potential lies within unravelling compositional changes of MPs during disease states [43]. Mass spectrometry-based analysis has a great potential to decipher the dynamic components of MP proteomes. Thus, Peterson et al. [44] showed that approximately 10% of the proteins are differentially expressed in endothelial cell-derived MPs from human umbilical vascular endothelial cells (HUVECs) depending on the activation stimuli [tumor necrosis factor (TNF) or plasminogen activator inhibitor (PAI)]. Similar observations have been made in cultured non-malignant and malignant cell-lines, monocytes, erythrocytes, and platelets. Bernimoulin et al. stimulated monocytic THP-1 cells with lipopolysaccharide (LPS) or a soluble P-selectin chimera [45]. Fifty-two and 408 proteins were uniquely expressed in the corresponding MP fractions. In particular, differential expression of proteins from subcellular Iocations such as mitochondria and adhesion molecules were enriched in MP-fractions from the soluble P-selectin chimera stimulated cells. Another study, comparing the proteome of MPs from platelets stimulated by either shear-stress or by thrombin, revealed that 26 proteins were differentially expressed [46]. These proteins were particularly related to cell assembly and organization and to cell morphology. In line with this, Milioli et al. [41] found that the platelet-derived MP protein content was highly dependent on the type of physiological agonist involved in platelet stimulation [thrombin, adenosine diphosphate (ADP), or collagen]. Interestingly, activation with stronger agonists resulted in platelet MP (PMP) overexpression of proteins related to platelet activation, while proteins involved in platelet degranulation and proteins from the electron transport chain were less abundant. In red blood cells, Prudent et al. [47] observed that calcium dependent proteins and annexins 4 and 5 were recruited in MPs generated by calcium stimulation compared to stored red blood cells (>40 days storage). In neutrophils, more than 400 distinct proteins could be identified in MPs released from neutrophils either in suspension (stimulated with fMLF, formyl-methionyl-leucyl phenylalanine), or adherent to a HUVEC monolayer, with only 223 proteins overlapping in the two MP-fractions [10]. Thus, mass spectrometry can uncover baseline and induced MP proteomes and pinpoint MP candidate biomarkers.

### Proteomics of human circulating microparticles identifies galectin-3 binding protein as a candidate biomarker for pathogenic microparticles

The human MP proteome studies to date have explored the proteome of the whole pool of plasma MPs, erythrocyte- (eMPs) or platelet-derived MPs (PMPs) isolated directly from plasma, or MPs generated in vitro from isolated platelets or granulocytes in healthy or diseased individuals [10–12, 37–41, 46, 48–56]. As can be seen from Table 1 the pre-analytical and analytical protocols differ between the studies on almost all points and caution regarding their interpretation is therefore warranted. The major observations are highlighted and put into perspective in the following sections.

#### Healthy individuals

Smalley et al. [38] observed that G3BP was more abundant in the total fraction of plasma MPs compared to the subfraction of platelet-derived MPs from healthy individuals. They also found an increased abundance of immunoglobulins, complement proteins, alpha-2-macroglobulin (A2M), and CD5 antigen-like protein in the total MP fraction. Together with this observation, the absence or very low abundance of G3BP in other proteomic studies of platelets, platelet- or erythrocyte-derived MPs during healthy conditions suggests that G3BP expression is a distinct feature of MPs derived from nucleated cells such as leukocytes and/or endothelial cells [37, 38, 41, 46, 49–53, 56]. In contrast to G3BP, A2 M was also found in erythrocyte-derived MPs and PMPs [52].

#### Systemic lupus erythematosus

SLE is an immune complex-mediated autoimmune disease linked to defective clearance of apoptotic cells, and circulating MPs are putative sources and traffickers of extracellular nuclear autoantigens. Østergaard et al. observed that circulating MPs from patients with SLE had increased quantities of G3BP compared to patients with systemic sclerosis and rheumatoid arthritis and to healthy controls [11, 57]. Ficolin-2, 14-3-3, β-2-glycoprotein-I, CD5 antigen-like protein, immunoglobulins, and complement proteins were also overabundant in SLE-MPs. Notably, A2M was not overexpressed. Overall, these findings suggested that more circulating MPs in SLE are of apoptotic origin and derive from leukocytes or endothelial cells, carry large quantities of autoantigens and immune complexes (ICs), and, finally, that G3BP is a distinguishing feature of such pathogenic MPs.

#### Venous thromboembolism

Ramacciotti et al. [12] found that G3BP was also enriched in plasma MPs in nine patients with deep venous thrombosis (DVT) compared to nine patients with leg pain without DVT and to six healthy controls. Also, complement proteins, immunoglobulins, A2M, CD5 antigen-like protein, clusterin, and polymeric immunoglobulin receptor were increased in MPs from DVT patients. It should be noted that in this study the samples were analysed three times and only A2M and G3BP were consistently identified in all three experiments in each patient. DeRoo et al. [58] explored the role of G3BP and galectin-3 (gal-3) in a mouse model of venous thromboembolism (VTE). Here they found G3BP in MPs, platelets, red blood cells, the thrombi and vein walls but not in leukocytes using quantitative immunoblotting and qRT-PCR. They did not observe a difference between the VTE and non-VTE mice regarding G3BP-MP quantities. Interestingly, significant levels of G3BP was found on the endothelium and bound to gal-3 on neutrophils facilitating their migration and extravasation. Gal-3 appeared to drive the vascular inflammation by stimulating the production of interleukin-6 and chemokine (C-C motif) ligand 2 (CCL2). This links both G3BP and gal-3 to inflammatory changes in VTE. In experimental VTE using four baboons, the same research group showed that fibrinogen and α_1_-anti-chymotrypsin were enriched and some immunoglobulins were down-regulated two days after a 6 h occlusion of the iliac veins [59]. G3BP was not enriched. The MP proteome in experimental rat VTE showed increased abundance of fibrinogens, macroglobulins including A2M, and immunoglobulins [60]. Thus, animal studies do not reproduce the findings of a G3BP-increase in human DVT; the interpretation of this discrepancy remains elusive.

#### Atherosclerosis

Several roles for MPs in the acceleration of atherosclerosis and arterial thrombosis have been proposed [4]. Mayr et al. [61] explored the proteome of MPs from atherosclerotic plaques. They found that the MPs were primarily of leukocyte origin based on the presence of surface markers (CD14, CD11c, CD18, CD51, and CD36), but that some also derived from smooth muscle cells and erythrocytes. Interestingly, immunoglobulins were also very abundant but primarily confined to the interior of the MPs, especially in MPs of macrophage origin, and the marked difference between antigen specificity compared to plasma immunoglobulins suggested a specific binding of antibodies to MP antigens [61]. Little et al. [39] characterized the plasma MP proteome in 42 individuals, the majority with a history of cardiovascular disease, hypertension, and diabetes. A core set of proteins was identified and correlated to gender and age, but, surprisingly, not to the other available cardiovascular risk factors. G3BP did not correlate with age and gender corroborating the utility of G3BP as an age/gender independent disease biomarker. Interestingly, at the same time the research group filed a patent application on G3BP as soluble biomarker for cardiovascular disease (patent application title and number: Galectin-3-Binding Protein as a Biomarker of Cardiovascular Disease, 20100055723) and recently observed that plasma G3BP correlated to mortality in coronary artery disease [62]. In patients with lacunar infarction, plasma MPs were characterized and correlated to clinical outcome and compared to healthy controls [55]. A differential expression of 43 proteins was observed. In particular, myelin basic protein was upregulated. G3BP and CD5 antigen-like protein were downregulated, while A2M showed minimal alterations. It should be noted that the MP isolation protocol differs significantly from the other protocols and most likely also includes a large proportion of exosomes (see Table 1 for details). Thus, based on a limited amount of data, an overexpression of G3BP on MPs in atherosclerosis or cardiovascular disease cannot be demonstrated.

#### A distinct overlap of the disease-associated microparticle proteomes in SLE and DVT patients

Despite the fact that so far only few and small studies with significant differences in methodology have been conducted, there is a striking overlap of MP-overexpressed proteins across the different disease entities, in particular G3BP, immunoglobulins, complement proteins, A2M, and CD5 antigen-like protein. Interestingly, G3BP and CD5 antigen-like protein were the only two common proteins found upregulated in plasma MPs, DVT, and SLE patient MP proteomes, while the common denominators in DVT and SLE were G3BP and A2M [11, 12, 38]. Accordingly, G3BP is the only consistently enriched protein across these three studies. Additionally, immunoglobulins and complement proteins were also found consistently enriched in the DVT and SLE MPs. SLE patients exhibit an increased platelet and coagulation activity and increased incidence of venous thrombosis. Altogether, these observations implicate MPs expressing these proteins as being involved in the disease pathogenesis and also suggest that these particular MPs originate from nucleated cells [10, 63, 64].

## Galectin-3-binding protein: structure, formation, and function

The formation, targets and biological functions of galectin-3-binding protein (also named Mac-2-binding protein) are yet not well defined. G3BP has been detected in several types of body fluids, such as serum, breast milk and semen, and is expressed in most tissues [18, 32]. In plasma, G3BP circulates in association with MPs as well as large soluble ring-like oligomers 30–40 nm in diameter with a molecular size larger than 1000 kDa [18, 39]. In vitro, G3BP is up-regulated by interferon (IFN)-α and -γ, viruses, and double-stranded polynucleotides [14, 23, 65]. Accordingly, plasma G3BP have been found elevated in diseases with increased IFN activity such as human immunodeficiency virus infection, chronic viral hepatitis, and SLE, but also in various types of solid cancers, Behcet’s disease, and rheumatoid arthritis [14, 66–72]. G3BP serves as a scavenger receptor which interacts with several targets [16]. It was originally identified (and named) in the search for gal-3 glycoprotein ligands [16, 17, 20]. Galectins are a group of 15 different carbohydrate binding proteins (lectins), termed galectin-1 (gal-1) to galectin-15 [13]. Gal-1 and gal-3 are involved in cell growth, adhesion, differentiation, inflammation, fibrosis, apoptosis, and metastasis [13]. Galectins bind via carbohydrate binding domains to galactosides on cell surfaces and on extracellular glycoproteins [64]. G3BP is heavy glycosylated and, as a major receptor for gal-3 and gal-1, it may be crucial for galectin mediated biological processes [18, 73]. Additionally, G3BP exhibits independent and selective binding to components of the extracellular matrix of the basement membrane in solid-phase assays, i.e. it interacts with collagen V and VI, fibronectin and nidogen, but not with laminin-1, fibulin-1 and -2, perlecan, and BM-40 [18]. G3BP also mediates cell–cell adhesion through binding to β1-integrin and LPS-dependently to CD14, and the protein seems to exert regulatory roles in immunity, e.g. through up-regulation of major histocompatibility class I (MHC-I) molecules and through the ability to stimulate natural killer (NK-) cells [18, 19, 32].

Collectively, G3BP is involved in the initiation and promotion of cell–cell adhesion and pro-inflammatory signalling cascades in viral infections, cancer (including metastasis), coagulation and haemostasis, and autoimmunity.

## The origin, role, and biomarker potential of G3BP-expressing microparticles

The proteomic studies of plasma MPs from disease entities characterized by some degree of intravascular cell death and activation combined with systemic and local inflammation, activation of the coagulation system, and thrombophilia suggest a common MP pathological proteome profile. Besides G3BP, these profiles also include immunoglobulins, complement proteins, A2M, and CD5 antigen-like protein. These observations suggest that MPs are tagged for removal during pathological states and that the overabundance of these proteins could reflect common upstream pathogenic processes such as increased cell activation and apoptosis in the circulating cells or endothelium along with activation of the coagulation and immune system.

### The cellular origin of G3BP-expressing microparticles

The cellular origin of G3BP-positive MPs under various conditions remains to be established. G3BP has been identified in high abundance in leukocytes, endothelial cells, and red blood cells and has been undetectable or present at a low abundance in platelets or platelet-derived MPs in humans. The G3BP gene expression is also dominant in type I IFN-activated neutrophils and peripheral blood mononuclear cells (PBMCs) in SLE patients [66]. In contrast, in a mouse model (C57CL/6 strain) G3BP was detected in platelets, circulating MPs, red blood cells, but not leukocytes [58]. This underscores the difficulty of translating animal studies of the molecular pathology of thrombosis (and SLE) to human systems. The available data from studies in humans strongly suggest that the majority of the G3BP-overexpressing MPs in the circulation originate from neutrophils, monocytes, and endothelial cells.

### The mechanisms behind microparticle G3BP-overexpression

G3BP overexpression on MPs most likely reflects the state of the parental cells, i.e. activation, stress, or, death. During these processes, G3BP is relocated into MPs and/or binds to upregulated MP surface molecules e.g. to gal-1 or gal-3. Generally, the composition and packaging of MPs is highly coordinated [7]. Increased G3BP associated with MPs may be caused by increased loading of G3BP during MP production in parent cells and tissues and/or increased binding of exogenous G3BP, e.g. from the circulation, to released MPs. As an example of differential expression, Dalli et al. only found low quantities of G3BP in MPs from fMLF-stimulated neutrophils and G3BP could not be detected in MPs originating from neutrophils stimulated by HUVECs. In contrast to the low G3BP expression generated by these stimuli, type I IFNs induce G3BP gene expression in vitro, and exploratory studies of IFN gene signatures in neutrophils and PBMCs from SLE patients identified G3BP as one of only few highly IFN-inducible genes [66, 74, 75]. However, it is unsettled if IFN activation results in increased G3BP in/on MPs, or, indeed, if systemic inflammation *per se* leads to increase in plasma-G3BP and a corresponding increase in binding of G3BP to MPs. Several studies suggest that increased MP-G3BP expression is part of the defence against viral infection [23, 74]. One possibility is that the virus-infected cells increase G3BP expression to eliminate viral particles by shedding G3BP-tagged MPs containing viral particles that are recognized and removed by professional phagocytes in the reticuloendothelial system. In our proteomic studies G3BP was the only IFN-inducible protein overabundant in SLE-MPs indicating that other mechanisms than type I IFN-induction controls MP-G3BP expression [11]. It is likely that G3BP overexpression simply reflects an increased number of apoptotic cell-derived circulating MPs in SLE patients. Lectins serve as sensors of apoptotic cells due to surface glycosylation changes on such cells. Accordingly, galectins significantly increase their binding to apoptotic cells, and G3BP subsequently binds to the galectins [63, 64]. In support of the notion of G3BP MPs being of apoptotic origin we found that G3BP is co-localized with glomerular immune complex deposits and on singular round structures in kidney biopsies from SLE patients [8]. As mentioned previously, apoptotic cells and MPs are major sources of extracellular autoantigens in the immune complex deposits, and the significant presence of G3BP in these deposits and on circulating MPs strongly corroborate the apoptotic origin of G3BP MPs [8, 76, 77]. Studies of the expression of G3BP on MPs from cells undergoing apoptosis or stimulated with IFN are needed to clarify the relative contributions of different cellular processes to the increase of G3BP-positive MPs in SLE and vascular disease.

### The role of G3BP-expressing microparticles in systemic lupus erythematosus

SLE is a systemic autoimmune disease capable of affecting most organ systems including the kidneys where glomerulonephritis (termed lupus nephritis, LN) is the major severe manifestation. Development of SLE is linked to defective clearance of apoptotic cells leading to an excess of highly auto-immunogenic cellular debris (apoptotic bodies and MPs) triggering anti-nuclear autoimmunity [8, 78–82]. An early important pathological feature is the occurrence of immune complex deposits in the glomerular basement membrane (GBM) that activate the complement system and incite inflammation [76, 83, 84]. Thus it is highly interesting to clarify the origin of these ICs in order to prevent their production and deposition and the subsequent inflammation and progression of LN. The origin of the ICs in the GBM is, however, unsettled. They may derive either from the circulation or locally in the glomeruli or even from a combination. The identification of G3BP as part of large MP-IC assemblies circulating in the blood of SLE patients is highly interesting in this context [57]. As mentioned, G3BP binds strongly to gal-1 and gal-3 and integrins, which are upregulated on endothelial cells, and also to basement membrane proteins [18]. Accordingly, G3BP may endow these MP-ICs with significant cell- and basement membrane adhesive properties in addition to affecting activation of immune cells. By binding to the endothelium and the extracellular matrix of the basement membrane G3BP may contribute to the MP-IC deposition and accumulation in the kidney [18, 19, 23, 32, 57, 85]. This may be aggravated by increased type I IFN-induced G3BP. Under normal conditions, G3BP may be part of the normal cell and MP clearance. However, the increased type I IFNs and G3BP in combination with a constant excess of highly altered immunogenic MPs may be crucial for the breakdown of tolerance and sustained systemic autoimmunity of SLE. Whether G3BP also could have adjuvant effects on the cellular arms of the immune system enhancing the stimulation by the MP-associated autoantigens is unclear. In any case, because of this multitude of possible pathogenetic mechanisms involving G3BP, this protein is an interesting putative therapeutic target [77]. By targeting G3BP the MP-autoantigenic “fuel” could be blocked reducing the IC deposition, activation of the complement system and the cellular arms of the immune system. This approach could prove more specific and thus less toxic and less prone to side effects than traditional immuno-suppressants.

### Galectin-3-binding protein and venous thromboembolism

VTE occurs as a result of blood stasis, hypercoagulability, and increased pro-coagulant adhesion molecules on endothelial cells in conjunction with inflammation. Circulating MPs are pro-coagulant and can contribute to thrombosis by their exposure of TF and phosphatidylserine [4]. Soluble, cell-, and MP-exposed gal-3 and G3BP may modulate thrombogenesis, particularly the involved inflammatory cascades, and are interesting future therapeutic targets [13].

It has been become apparent that inflammation contributes to VTE. In a study by deRoo and coworkers, activated platelets expressed P-selectin and PSGL-1 that lead to increased leukocyte adhesion and thrombus formation. Binding of G3BP to gal-1 on the platelets resulted in an increased P-selectin expression and promoted platelet activation and thrombogenesis [58]. This may trigger increased MP-G3BP release and perpetuate the platelet activation. Also, G3BP and gal-3 affected the cell–cell adhesion on the thrombus-endothelial interface and the thrombus itself and were increased on the endothelium in a mouse model of VTE [58]. Additionally, in this study, during early VTE gal-3 was greatly increased and G3BP and gal-3 co-localized in the leukocyte/endothelial cell interface and leukocytes were attached to the vein wall. The thrombus size correlated with gal-3 and interleukin-6 in the vein wall, and the vein wall inflammatory changes seemed driven by triggering of IL-6 by gal-3 and the pro-inflammatory chemokine CCL2. In patients with acute VTE elevated levels of circulating G3BP were found. Accordingly, G3BP seems to play a role in the pathogenesis of VTE and thrombophilia. In SLE increased rates of VTE are a prominent feature and G3BP may be a novel thrombophilia risk factor independent of the presence of anti-phospholipid antibodies. We have also observed that increased levels of plasma G3BP were a predictor of venous thromboembolic events in SLE patients independent of the presence of anti-phospholipid antibodies (unpublished data). Clarification of the role of G3BP needs further exploration in VTE. If further studies support an important role for G3BP in thrombosis the development of G3BP or gal-3 blocking compounds seems to be attractive future therapeutic approaches.

### G3BP-expressing microparticles as future biomarkers

Generally, in inflammatory and thrombotic diseases G3BP-overexpressing MPs appear to be promising diagnostic or prognostic biomarkers as well as biomarkers of disease activity. Additionally, G3BP-MPs could be sensitive novel biomarkers of apoptotic activity relevant in autoimmunity, cardiovascular disease, and cancer.

Specifically, MP-associated G3BP may represent several advantages compared with soluble plasma G3BP used as biomarkers. Total plasma G3BP and G3BP-MPs levels do not completely mirror each other [14]. This suggests that G3BP is differentially expressed in different compartments in the blood. Mass spectrometry provides a global measure of G3BP in a whole pool of pelleted MPs. Different populations of G3BP-positive MPs based on G3BP and PS surface densities can be discerned in the circulation in both healthy individuals and SLE patients using flow cytometry [14]. Only one of the flow cytometrically identified G3BP-positive populations correlated with plasma G3BP levels, while there were no apparent correlation between plasma G3BP and the MP G3BP quantities obtained by mass spectrometry. In SLE, G3BP-MP expression distinguished SLE patients from healthy controls and from patients with other autoimmune diseases (systemic sclerosis and rheumatoid arthritis) and it seems worthwhile to test the diagnostic potential in larger prospective cohorts of patients presenting with signs of systemic connective tissue disease [11]. In relation to disease activity, clinical, or immunological manifestations, there was no correlation to the G3BP-MP quantities obtained by mass spectrometry [14]. The concentrations of one of the flow cytometrically identified sub-populations (G3BP and annexin V positive) correlated to anti-dsDNA levels but not to disease activity or to clinical or serological manifestations [8]. This links G3BP-expressing MPs to polyclonal activation of plasma cells or the effects thereof, but their use as biomarkers of disease activity seems limited in SLE.

In VTE increased levels of d-dimer are sensitive markers of VTE, but are, however, not specific. G3BP-MPs may contribute to the specificity, perhaps as part of an MP-DVT profile with A2M and CD5 antigen-like protein. In VTE little focus has been on monitoring, damage and outcome and a role for G3BP as a biomarker here is difficult to predict.

Plasma levels of G3BP may reflect macrophage activity and type I IFN activity [14, 15]. To a certain degree this may also be the case for G3BP-expressing MPs. Type I IFNs are associated with subclinical markers of atherosclerosis in vitro [86, 87]. Thus it interesting that the plasma concentrations of G3BP are significantly associated with the degree of subclinical atherosclerosis in patients with HIV and hepatitis C [15]. This may also be the case in SLE, where increased type I IFNs and accelerated atherosclerosis are dominant features. Recently, plasma G3BP levels were independently associated with long-term cardiovascular mortality [62]. Little et al. [39] do not report on the putative correlations between the G3BP MP quantities and cardiovascular comorbidities, which would be interesting area to explore for future atherosclerosis biomarker studies.

## Conclusions

The unbiased dissection of the circulating MP proteome has led to novel discoveries of candidate MP proteins associated with venous thrombosis and systemic lupus erythematosus. These include G3BP, immunoglobulins, complement, A2M, and CD5 antigen-like protein. In general, the candidate MP biomarkers provide new tools to explore the roles of MPs in disease pathogenesis and as biomarkers. The restricted number of differentially expressed MP proteins fit the notion that the proteins reflect the specific state of their parental cells. Accordingly, the MP-proteins serve as biomarkers for MPs that either reflect or have direct roles in the disease pathogenesis, i.e. patho-phenotypical or pathogenic MPs, respectively. In SLE, high IFN-activity and improper clearance of apoptotic cells may lead to the overabundance of apoptotic-derived MPs with high expression of G3BP that form large circulating immune complexes. G3BP on the surface of the MPs most likely facilitates the deposition of the MP-ICs in the kidney promoting glomerulonephritis. MP-G3BP may thus have a direct role in SLE pathogenesis, while also serving as a biomarker in lupus nephritis, and of circulating apoptotic-derived MPs. While the proteomic studies clearly associate MP-G3BP overexpression with SLE and deep venous thrombosis, we anticipate that the future potential of G3BP and the other MP candidate biomarkers may be even far greater in cancer and chronic viral diseases. A major limitation of the proteome discovery studies is often the small number of patients and controls. With the identification of the MP-biomarkers, the proteins can now be easily incorporated in large MP-biomarker panels together with cell-specific markers, annexin V, or other MP molecules of interest enabling high-throughput targeted MP studies in large cohorts of patients. Such large studies are now feasible and highly warranted in the fields of coagulation and hemostasis, infection, cancer, and autoimmunity.

## Abbreviations

1-D: one-dimensional
2-D: two-dimensional
ADP: adenosine diphosphate
A2M: alpha-2-macroglobulin
CCL2: chemokine (C-C motif) ligand 2
CD: cluster of differentiation
DNA: deoxyribonucleic acid
eMPs: erythrocyte-microparticles
fMLF: formyl-methionyl-leucyl phenylalanine
Gal-3: galectin-3
G3BP: galectin-3-binding protein
GBM: glomerular basement membrane
HUVECs: human umbilical vascular endothelial cells
ICAT: isotope-coded affinity tag
Ics: immune complexes
IFN-α: interferon-α
LC–MS/MS: liquid chromatography-tandem mass spectrometry
iTRAQ: isobaric tags for relative and absolute quantitation
LPS: lipopolysaccharide
LN: lupus nephritis
MHC: major histocompatibility class
MALDI-TOF: matrix-assisted laser desorption/ionization time of flight
MVs: microvesicles
MPs: microparticles
mRNA: messenger RNA
miRNA: microRNA
MS: mass spectrometry
NK: natural killer
PAI: plasminogen activator inhibitor
PBMCs: peripheral blood mononuclear cells
PMPs: platelet-derived MPs
RNA: ribonucleic acid
SLE: systemic lupus erythematosus
TF: tissue factor
TNF: tumor necrosis factor
VTE: venous thromboembolism
